# Why is alcohol so normalised in Europe?

**DOI:** 10.1016/j.lanepe.2024.101119

**Published:** 2024-11-01

**Authors:** The Lancet Regional Health – Europe

Eight of the ten countries with the highest global alcohol consumption per-capita are located in the WHO European Region. Considering this level of consumption, it is not surprising that the Region has the highest number of alcohol-attributable deaths per 100,000 persons accounting for nearly one in 11 of all deaths.

Alcohol consumption is causally linked to more than 200 diseases, most commonly liver disease, several cancers, cardiovascular disorders, and mental health conditions. Alcohol is a key contributor to violence, including sexual and gender-based violence, road traffic injuries, financial instability, and social problems.

Despite its well-documented harms, such as health impacts and societal costs, and the absence of any safe level of consumption regarding cancer risk, alcohol use remains deeply embedded in European society. This raises profound questions about why the consumption of such a harmful substance continues to be normalised.

A key reason is that alcohol is often perceived as an important part of traditions, celebrations, and social rituals. The cultural acceptance of alcohol use is passed down through generations, making it an almost unquestioned staple of social interaction. In societies where alcohol consumption is the norm, social pressure to conform sustains its prevalence, with peer approval often shaping consumption patterns, particularly among younger adults.

The persistence of a practice doesn't justify its continuation—just because it has been ingrained in society for generations doesn't mean cultural norms cannot change. As demonstrated with tobacco, cultural norms can and do shift over time.

The second key issue is the lack of public awareness of alcohol-related harms. For example, only half of people in the European Union (EU) are aware that alcohol can cause cancer, and knowledge about the specific types of cancer it can cause is even lower. Additionally, widespread misinformation persists, with many people believing that alcohol has supposed health benefits, such as for heart health. Others consume alcohol to relax, relieve stress, or for social connection, which reinforces regular use while masking the long-term risks.

Third, and a major driver of pervasive alcohol use, is the influence the alcohol industry exerts through aggressive marketing and lobbying, including corporate political activity. Alcohol is often portrayed as glamorous, fun, and socially essential in marketing campaigns, and lobbying efforts tend to frame alcohol as a complex issue, where the causal links to harm are difficult to establish and emphasise that its use is only problematic for a minority of heavy drinkers. Such strategies create doubt, undermine public health campaigns, and downplay the associated risks.

The global alcoholic beverages market size reached approximately US$1.75 trillion in 2023, a figure that exceeds the gross domestic product of many countries. This figure is expected to grow, with forecasts predicting the market could reach $2.34 trillion by 2032. The sheer scale of the alcohol industry demonstrates the considerable financial influence the alcohol industry exerts globally, further complicating efforts to regulate alcohol use and mitigate its public health impact.

To effectively raise awareness of alcohol-related harms, it is crucial to dissociate alcohol from cultural traditions, strengthen public health campaigns, limit alcohol sales, and curb the influence and aggressive marketing of the alcohol industry.

Research has shown that robust awareness initiatives such as health warnings and labelling on packaging have proven successful in educating people about the risks of alcohol use. Larger warnings and graphic images on tobacco products have successfully reduced consumption in the past 30 years. However, similar efforts for alcohol have received substantial resistance from the alcohol industry and governments.

As of 2024, only 13 of 53 WHO European Region Member States mandate health warnings on alcohol labels, with just three EU Member States (France, Germany, and Lithuania) currently enforcing this requirement, and mandating health warnings on all sold alcoholic beverages from May, 2026, onwards. Despite alcohol labelling being prioritised in both the European Framework for Action on Alcohol 2022–2025 and WHO's Global Alcohol Action Plan 2022–2030, warnings remain poorly implemented. Turkmenistan is the only country with a general health warning, while most countries only target specific groups at risk of alcohol-related harms (eg, young people or pregnant women), or use terms such as excessive use or alcohol misuse, which minimise the perceived risk. Alcohol industry lobbying often influences these messages, shifting responsibility onto individuals with the aim of protecting their business interests and normalising the use of alcohol in society.

Current trends indicate that WHO's global target to reduce harmful alcohol use by 20% by 2030, compared with 2010 levels, will not be met. In view of the observed revenue growth within the alcohol industry, the alcohol-related harms are likely to increase.

In addition to public health measures, it is crucial for health institutions, research organisations, and advocates to lead by example. To truly drive down alcohol consumption, we must embody the change we seek. A simple, yet powerful step would be to eliminate the serving of alcohol at scientific and global public health conferences. This move would send a clear, values-driven message: public health cannot be compromised for tradition. By reshaping the culture around these events, we can foster environments that prioritise health and wellbeing over social norms that perpetuate unhealthy behaviours.

